# Quick and Easy Application Method of TachoSil®️ During Laparoscopic and Robotic Liver Resections

**DOI:** 10.7759/cureus.37252

**Published:** 2023-04-07

**Authors:** Masatoshi Kajiwara, Shigetoshi Naito, Takahide Sasaki, Ryo Nakashima, Suguru Hasegawa

**Affiliations:** 1 Gastroenterological Surgery, Faculty of Medicine, Fukuoka University, Fukuoka, JPN

**Keywords:** hemostasis, laparoscopic liver resection, robotic liver resection, liver parenchymal transection, liver surgery, tachosil®️

## Abstract

TachoSil®️, a fibrin sealant patch, is a sheet-type hemostatic agent. Therefore, it is technically demanding to put it on the target place especially in laparoscopic surgeries due to the motion restriction of straight-fixed instruments. This article describes a quick and easy technique of TachoSil application during laparoscopic liver surgeries, by sewing it to the laparoscopic gauze in advance. This method allows for one-handed operation and stress-free application even in the situation of active bleeding.

## Introduction

Secure hemostasis is the most important step during liver surgeries. TachoSil®️ (CSL Behring, K.K., Tokyo, Japan) is a topical fibrin sealant patch and one of the reliable hemostatic agents during liver parenchymal transection [1-3]. TachoSil is a sheet-type hemostatic agent, and applying it to the targeted place is quite easy in the case of open liver surgery. It becomes, however, technically challenging during laparoscopic hepatectomy due to the motion restriction of straight-fixed instruments.

To prevent TachoSil from adhering to places other than the target site, it seems to be common to wrap it in a paper sheet or laparoscopic gauze and carry it to the liver surface. However, in this method, both hands are required for manipulation, it is time-consuming, and in addition, it sometimes becomes a mess and useless by sticking to the forceps.

To solve this problem, we sewed TachoSil onto the laparoscopic gauze in advance, which enabled the delivery of TachoSil to the bleeding point with one hand, realizing accurate and stress-free application. This article describes this quick and simple technique with videos.

## Technical report

**Figure 1:**
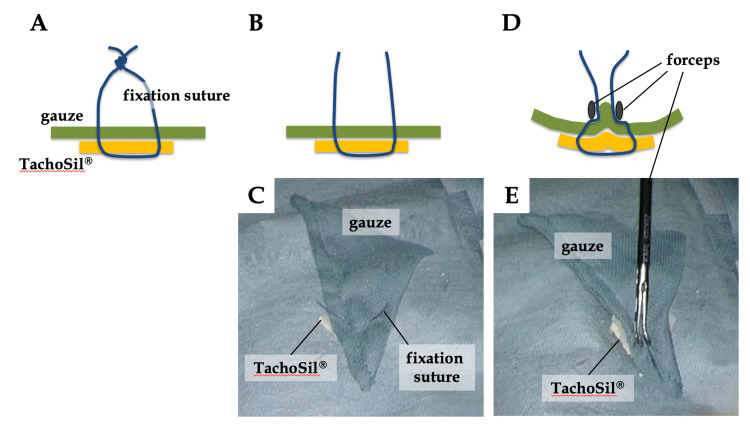
TachoSil®️ was sewn onto the laparoscopic gauze in advance. (A) Original version with a ligation. (B and C) Current version without a ligation. (D and E) Even without a ligation, TachoSil®️ and the gauze are not displaced if both the suture and gauze are securely grasped with the forceps.

A typical setting of laparoscopic liver resection was described previously [4]. Forty-eight millimeter (1.9 inches) square TachoSil is cut crosswise into four equal pieces and stitched to the laparoscopic gauze with the white side of TachoSil (non-active side) inside (Video 1). Originally, the fixation suture was tied, but now it is not (Figures 1A-1C). Even without a ligation, TachoSil and the gauze are not displaced if both the suture and gauze are securely grasped with the forceps (Figures 1D, 1E). It has become more convenient because the step of tying the thread in the preparation stage and cutting it inside the abdomen has been eliminated. TachoSil with the gauze is easily delivered through a 12 mm port (Video 1).

**Video 1:** Preparation TachoSil®️ was sewn onto the laparoscopic gauze in advance.

After removing the fixation suture, TachoSil is pressed against the bleeding site while moistening the gauze with saline not to stick to TachoSil. TachoSil is held against the liver surface for a few minutes to ensure uniform contact.

Case 1: laparoscopic S7 partial resection

Laparoscopic S7 partial resection was performed for suspected hepatocellular adenoma. During the removal of the specimen from the abdominal wound, the hematoma accumulated at the liver resection surface. The bleeding point was revealed to be close to the inferior vena cava, and TachoSil was applied (Video 2).

**Video 2:** Laparoscopic S7 partial resection During the removal of the specimen from the abdominal wound, the hematoma accumulated at the liver resection surface. The bleeding point was revealed to be close to the inferior vena cava (IVC) and TachoSil®️ was applied.

Case 2: robotic S7 segmentectomy

Robotic S7 segmentectomy was performed for hepatocellular carcinoma. After the specimen was removed, TachoSil was delivered through a 12 mm assistant trocar and attached to the stump of the Glissonean pedicle of Segment 7 (G7) (Video 3).

**Video 3:** Case 2: Robotic S7 segmentectomy After robotic S7 segmentectomy, TachoSil®️ was delivered through a 12 mm assistant trocar and attached to the stump of Glissonean pedicle of Segment 7 (G7). After removing the fixation suture, TachoSil was pressed against the stump of G7 while moistening the gauze with saline not to stick to TachoSil.

This method is useful in robotic liver resection as well as laparoscopic liver resection.

Case 3: laparoscopic S8 segmentectomy

Laparoscopic S8 segmentectomy was performed for a metastatic liver tumor from rectal cancer. Two bleeding points were observed in the branches of the right hepatic vein just as the hepatectomy was completed. About 1 cm square TachoSil was applied (Video 4).

**Video 4:** Case 3: Laparoscopic S8 segmentectomy After laparoscopic S8 segmentectomy, two bleeding points were observed in the branches of the right hepatic vein (RHV). About 1 cm square TachoSil®️ was applied. This was an original version, and the tie needed to be cut.

 The size of TachoSil can be adjusted according to the degree and location of bleeding. This was an original version, and the tie needed to be cut.

## Discussion

Although oozing from the liver resection surface is usually controllable by pressure alone, other hemostatic techniques are required in the case of active bleeding. The major bleeding from hepatic vein injury is sometimes tried to be controlled by stitches, but there is a risk that the suturing will enlarge the bleeding point if the vein wall is thin or fragile. Considering this, TachoSil, a sheet-type hemostatic product, is a good indication for controlling bleeding from the hepatic vein, which is a low-pressure system [1-3].

However, the utility of TachoSil during laparoscopic liver resection remains poorly analyzed mainly because laparoscopic positioning of patching agents on the resection surface is technically demanding [5]. No detailed reports on how to apply TachoSil laparoscopically were available. As TachoSil is fragile and sticky and needs to be handled carefully, it takes time and practice to manipulate a small piece of TachoSil during laparoscopic surgeries using straight-fixed instruments with both hands. 

Our method allowed for one-handed insertion and application of TachoSil, relieving stress caused by operational restrictions. In addition, since it has been confirmed that even a small size of about 1 cm square TachoSil can be applied, our method may contribute to medical cost reduction [6].

However, this method requires a flat liver resection surface to some extent for attaching a sheet-type TachoSil. Therefore, a gel-like hemostatic agent such as thrombin glue may be more suitable for bleeding from the bottom of the resected liver. To prevent rebleeding after the liver resection surface, a powdered hemostatic agent (e.g., oxidized regenerated cellulose powder) is ideal to evenly cover a wide resection area at the end of the surgery.

As described above, it is important to select and appropriately use the most suitable hemostatic agent according to the purpose of hemostasis while considering the state of the liver resection surface and the degree of bleeding [7]. Although we have not actually tried it yet, this method could be useful for venous bleeding during other types of surgeries such as laparoscopic or robotic pancreatic resection.

## Conclusions

We reported a simple and quick TachoSil application technique during laparoscopic and robotic liver surgeries. This stress-free method can be operated with one hand. Therefore, it is applicable even in the situation of active bleeding. Additionally, it is possible to use TachoSil as small as 1 cm square and is expected to be superior in terms of surgical cost. Laparoscopic and robotic liver resections are becoming more common, and expanding the options for safe hemostasis is critical for the spread of safe and secure hepatectomy.
